# A randomized controlled trial of botulinum toxin A for treating neuropathic pain in patients with spinal cord injury: Retraction

**DOI:** 10.1097/MD.0000000000007871

**Published:** 2017-08-18

The article, “A randomized controlled trial of botulinum toxin A for treating neuropathic pain in patients with spinal cord injury”,^[1]^ which appeared in Volume 96, Issue 20 of *Medicine*, has been retracted for plagiarism and academic fraud. Similar design study, language, and tables were found in a previously published article, “Botulinum toxin type A for neuropathic pain in patients with spinal cord injury”,^[2]^ which appeared in Volume 79, Issue 4 of *Annals of Neurology*, without any attribution or citation.
